# Cutaneous leishmaniasis by a needlestick injury, an occupational infection?

**DOI:** 10.1371/journal.pntd.0011150

**Published:** 2023-03-08

**Authors:** Alejandra Perales-González, Daniela Michelle Pérez-Garza, Valeria Fernanda Garza-Dávila, Jorge Ocampo-Candiani

**Affiliations:** Department of Dermatology, Hospital Universitario “Dr. José Eleuterio González,” Universidad Autónoma de Nuevo León, Monterrey, México; National Institute of Allergy and Infectious Diseases, UNITED STATES

## Abstract

Leishmaniasis is a parasitic disease caused by over 20 species of *Leishmania*. Transmission is mainly via sandfly bites infected with promastigotes, through the placenta from mother to child, by sexual intercourse, blood transfusion, and occupationally acquired by direct inoculation into the skin. Clinical manifestations vary from self-limited cutaneous disease to a life-threatening visceral infection. In November 2021, a 29-year-old otherwise healthy dermatology resident suffered an accidental needlestick injury while performing a biopsy on a patient with a presumptive diagnosis of an infectious dermatosis, later confirmed as mucocutaneous leishmaniasis caused by *Leishmania panamensis*. Later, the resident developed an erythematous, painless papule at the point of inoculation, with a central ulcer and painful enlargement of ipsilateral lymph nodes. Biopsy was compatible with leishmaniasis. After completing a 20-day treatment with meglumine antimoniate, the ulcer had healed completely. At the 6-month follow-up, both patients remain asymptomatic. This case serves as a reminder that health providers should have the proper training and knowledge of their hospital management protocol for occupational injuries. Moreover, physicians should bear in mind that leishmaniasis is not exclusively transmitted by sandfly vectors.

## Author summary

Leishmaniasis is a parasitic disease usually transmitted by sandfly bites, but also by placental transmission, sexual intercourse, blood transfusion, and direct inoculation into the skin. Clinical manifestations range from self-limited cutaneous disease to life-threatening visceral infection. Healthcare workers who come into contact with patients and/or their body fluids are at risk for occupational infection. The authors present the case of a healthcare provider who suffered an accidental autoinoculation with a needle contaminated with Leishmania organisms. When encountering cutaneous leishmaniasis, physicians should remember that leishmaniasis can also be transmitted by direct inoculation in a clinical setting and take contact and needle precautions. In the event of an accidental puncture, knowledge of the morphology and natural history of cutaneous leishmaniasis may be critical for diagnosis and thus prompt treatment. Therefore, education on the principles and practices to avoid transmission of infectious diseases should begin during training.

## Introduction

Leishmaniasis is a parasitic disease caused by over 20 species of *Leishmania*. Transmission is mainly via sandfly bites infected with promastigotes [1]. Other reported transmission mechanisms are vertical transmission through the placenta from mother to child, by sexual intercourse, blood transfusion, and occupationally acquired by direct inoculation into the skin [2]. Clinical manifestations vary from self-limited cutaneous disease to a life-threatening visceral infection, depending on the parasite species, vector, biology, and host’s immune response [1]. We present a case of an accidental direct inoculation of *Leishmania panamensis*.

## Case presentation

**Fig 1:**
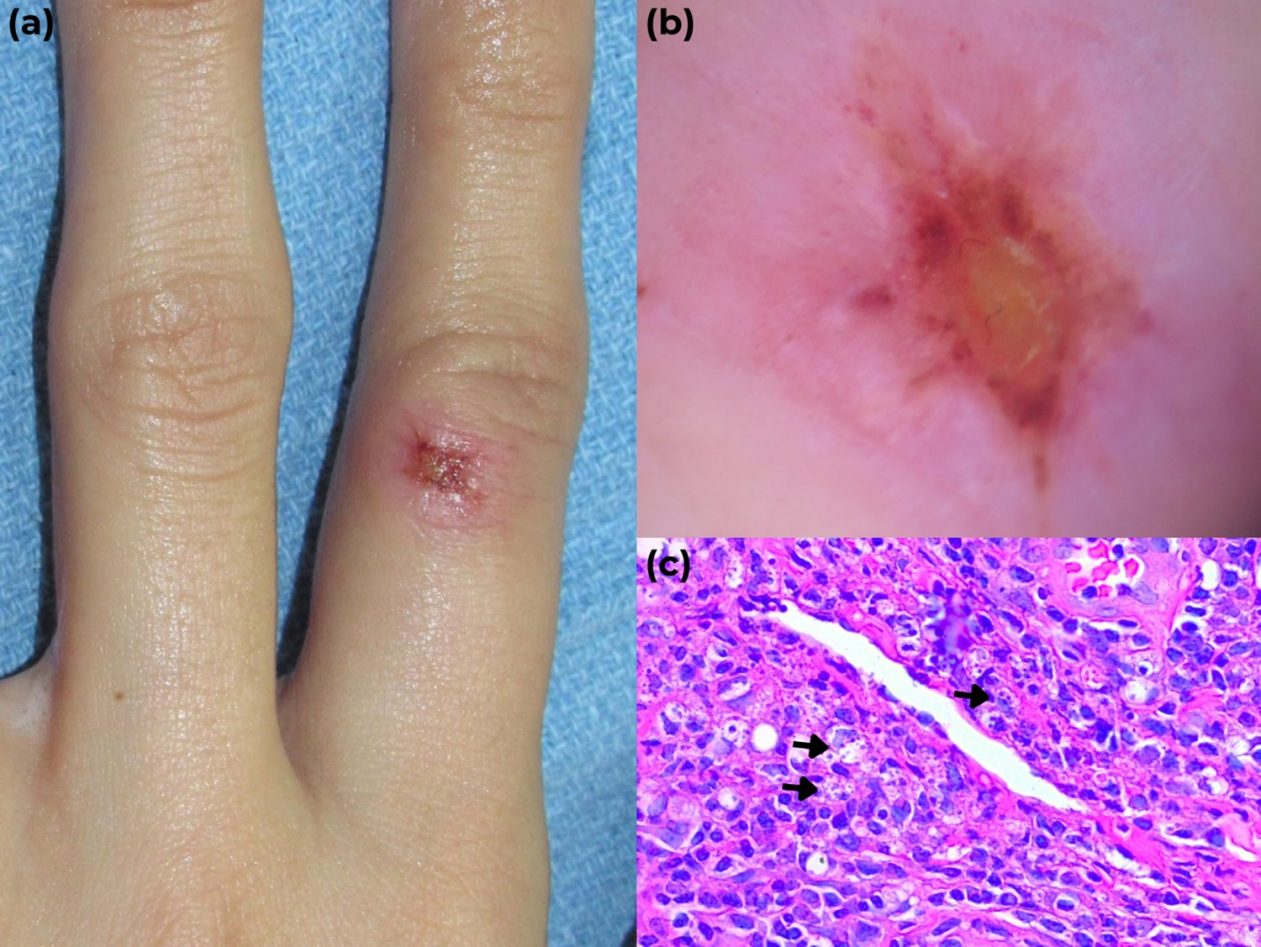
(a) Erythematous papule with central ulceration on the proximal phalanx of the left index finger, at the puncture site before treatment. (b) Dermoscopy shows erythema with a yellowish hue, central ulceration, peripheral white lines, and dotted vascular structures. (c) Histologic findings evidenced a dermal lymphohistiocytic infiltrate with amastigote organisms within histiocytes (hematoxylin & eosin; original magnification ×40).

In November 2021, a 29-year-old otherwise healthy dermatology resident treated a Haitian immigrant patient with a presumptive diagnosis of an infectious dermatosis. She performed a skin biopsy on the patient and suffered an accidental needlestick injury on the left index finger. The protocol for the management of needlestick and sharps-related injuries was implemented; blood tests, including a panel for HIV, hepatitis B, and hepatitis C virus, had no significant findings. The Haitian patient was diagnosed with mucocutaneous leishmaniasis caused by *Leishmania panamensis*, and treatment was established with liposomal amphotericin B at 3 mg/kg per day intravenously (IV) for 10 days, followed by meglumine antimoniate at 20 mg/kg per day IV for 28 days, with complete remission. In January 2022, 2 months after the puncture, the resident developed an erythematous, painless papule at the point of inoculation (Fig 1A). Dermoscopy showed an erythematous background with a yellowish hue, central ulceration, peripheral white lines, and dotted vessels (Fig 1B). Over 3 weeks, the lesion worsened, increased in size, and presented a central ulcer and small satellite papular lesions. Painful enlargement of ipsilateral lymph nodes along her left forearm was also noted. A skin biopsy was compatible with leishmaniasis (Fig 1C). A 20-day treatment with meglumine antimoniate, at 20 mg/kg per day IV, was established. After completing treatment, a small defect remained, so three additional doses of 1 ml intralesional meglumine antimoniate were administered. A week after the last injection, the ulcer had healed completely. At the 6-month follow-up, both patients remain asymptomatic.

## Discussion

Leishmaniasis is a neglected tropical disease that mainly affects people from underdeveloped countries in Africa, Asia, and Latin America. According to the World Health Organization (WHO), around 700,000 to 1,000,000 new cases occur annually. It is associated with malnutrition, population displacement, poor housing, unsanitary conditions, a weak immune system, and a lack of financial resources [3].

Although the most common transmission mechanism is by female sandflies’ bite, cases of direct cutaneous inoculation have been reported in the literature [4–7], thereby making it an occupational infection. Occupational infections are defined by a particular infectious agent or organism associated with an occupational setting and specific work activities that predispose to exposure and, therefore, to the infection [8].

Healthcare workers who are in contact with patients and/or their body fluids are at risk of occupational infections with subsequent risk of contracting diseases, disability, and even death [9]. Education towards the principles and practices for the prevention of the transmission of infectious disease should begin during training in healthcare professionals. The Centers for Disease Control and Prevention has guidelines for preventing the transmission of infectious diseases in the healthcare workplace, including needlestick and other sharps-related injury prevention programs [10]. No specific statements are made for leishmaniasis.

When encountering cutaneous leishmaniasis, healthcare workers should keep this mechanism of transmission in mind and implement contact and needle precautions. When suspecting an infectious etiology, skin biopsies of ulcerated lesions should be allowed to heal by secondary intention rather than suturing since friable tissue could easily tear and cause poor control of needles and other sharps. Furthermore, in the case of an accidental puncture, bearing in mind the morphology and natural evolution of cutaneous leishmaniasis could be crucial for diagnosis and, thus, prompt treatment.

## Conclusions

Here, we present the case of a healthcare provider who suffered from an accidental autoinoculation with a needle contaminated by *Leishmania* organisms. This case serves as a reminder that health providers should have the proper training and knowledge of their hospital management protocol for occupational injuries. Moreover, physicians should remember that leishmaniasis is not exclusively transmitted by sandfly vectors but also by direct inoculation in a clinical setting.

## Ethics statement

Informed written consent was obtained for clinical photographs.
